# Enhanced photo-sensitivity through an increased light-trapping on Si by surface nano-structuring using MWCNT etch mask

**DOI:** 10.1186/1556-276X-6-573

**Published:** 2011-10-31

**Authors:** Min-Young Hwang, Hyungsuk Kim, Eun-Soo Kim, Jihoon Lee, Sang-Mo Koo

**Affiliations:** 1 College of Electronics and Information, Kwangwoon University, Nowon-gu Seoul 139-701, South Korea; 2 Institute of Nanoscale Science and Engineering, University of Arkansas, Fayetteville, AR 72701, USA

## Abstract

We demonstrate an enhanced photo-sensitivity (PS) through an increased light-trapping using surface nano-structuring technique by inductively coupled plasma (ICP) etching on multi-walled carbon nanotube (MWCNT) etch masked Si with hexamethyl-disilazane (HMDS) dispersion. In order for a systematic comparison, four samples are prepared, respectively, by conventional photolithography and ICP etching using MWCNT as a etch mask. MWCNT-etched Si with HMDS dispersion shows the highest RMS roughness and the lowest reflectance of the four. Two test device structures are fabricated with active regions of bare-Si as a reference and MWCNT etch masked Si with HMDS dispersion. The increased light-trapping was most significant at mid-UV, somewhat less at visible and less noticeable at infrared. With an ICP-etched Si using CNT HMDS dispersion, PS is very sharply increased. This result can lead to applications in optoelectronics where the enhancement in light-trapping is important.

## Introduction

Light-trapping, in other word optical absorption, in such device applications as photovoltaics, light-emitting diodes, light sensors, photo-diodes, and transistors, plays an important role in their device functionality and in order to suppress reflection losses and increase conversion efficiency [1-10]. In general, approximately 30-40% of photons are reflected when incident on planar wafers. Theoretically, through an ideal light-trapping the length of optical path in a material with a refractive index of *n *can be enhanced by a factor of 4*n*^2 ^[11], and thus the amount of photons that can be absorbed in a material can be significantly enhanced. Various light-trapping techniques therefore have been explored and developed to restrain the reflection losses and enhance optical absorption in various applications. For example, in photovoltaic applications a thin film known as an antireflection coating can be adapted, which has a refractive index that is intermediate between those of semiconductors (*n*_s_) and air (*n*_0_) [11]. TiO_2 _(*n *= 2.3), Ta_2_O_5 _(*n *= 2.25), Si_3_N_4 _(*n *= 2.0), Al_2_O_3 _(*n *= 1.85), SiO_2 _(*n *= 1.5), and MgF_2 _(*n *= 1.38) are widely known materials that can be used for antireflection coatings [8]. On the other hand, surface patterning or texturing instead of planar substrates have been widely investigated and adapted in order to handle light-trapping in a more efficient way [12-14]. Patterns that can be used in surface texturing can be either regular or random. A regularly textured surface can be yield with various types of patterns [3,5,6] using conventional photolithography. Randomly textured surfaces have been demonstrated [4,7-10] using SnO_2_, ZnO, Ag, glass, and plastics, which showed an improved spectral response in longer wavelengths. Meanwhile, CNTs have been widely proposed in composite materials to reinforce the mechanical strength and catalytic activities [15-21], which varies from metals, metal oxides, and ceramic composites to polymers. CNTs can also be used in various applications such as an emitter for the field effect displays, micro-supercapacitors, color fine-tuning, electrochemical sensors and hydrogen storage, etc. [22-29]. On the other hand, due to their superior mechanical strength, CNTs can be used as a etch mask in plasma dry-etching process [30]. While etch masks through conventional photolithography process can generate micron-scale patterns, CNT etch mask technique can provide nanoscale surface patterns as observed by microscopy [31-33].

In this letter, we demonstrate an enhanced photo-sensitivity (PS) through an increased light-trapping on Si, which is achieved by increasing surface roughness and suppressing reflection losses through a surface nano-texturing using inductively coupled plasma (ICP) etching. Four Si samples are prepared using conventional photolithography and ICP etching using multi-walled carbon nanotubes (MWCNT). MWCNTs dispersed in hexamethyl-disilazane (HMDS) were used as an etch mask for the ICP etching. The highest RMS roughness with a value of 7.66 nm and thus the lowest reflectance by 41.5% at mid-UV are achieved with the ICP-etched Si using MWCNT etch mask. The increased light-trapping is most significant at mid-UV, less at visible, and finally somewhat insignificant at infrared region. Based on the RMS roughness and reflectance analyses, two *I*-*V *test device structures are fabricated. Device active region using bare-Si is set as a reference while ICP-etched Si using MWCNT etch mask is fabricated for a PS comparison. With an ICP-etched Si with HMDS dispersion, PS at UV illumination is very sharply increased through back-to-back Schottky-barriers.

### Experimental details

In this experiment, in order to perform surface morphology, RMS roughness, and reflectance analyses, four Si samples were prepared: bare-Si (sample A), square trench-patterned Si using conventional wet chemical etching (sample B), ICP-etched Si using MWCNT-dispersion in isopropyl-alcohol (sample C), and another ICP-etched Si using MWCNT-dispersion in HMDS (sample D). For the preparation of sample A, a conventional cleaning procedure using acetone and methanol was performed. For sample B, a pattern area of 3 × 3 μm^2 ^was fabricated using conventional photolithography and wet chemical etching; namely chemical cleaning using H_2_SO_4_:H_2_O_2 _= 1:1 and HF, photo-resist (PR) spin coating, baking, UV exposure and development, etc. After the patterning, 50 nm of Si was removed using HNO_3_:HF:DI = 100:3:40. For the preparation of ICP-etched Si, MWCNTs (MWCNTs) with a diameter of 10-15 nm were used (Hanhwa nanotech Co., Korea), which were grown using a chemical vapor deposition. For the preparation of one ICP-etched Si sample (sample C), initially approximately 13.5 mg of MWCNTs was dispersed in approximately 200 mL of isopropyl-alcohol. The mixed solution was then dropped on a Si surface and the sample was dried in air. Subsequently, the sample was heated on a hot-plate at 120°C for 2 min to fix the MWCNTs. For the preparation of the other ICP-etched Si (sample D), MWCNTs were dispersed in HMDS for an improved dispersion. For the samples C and D, 50 nm of Si was subsequently etched away using ICP dry-etching with an ambient gas mixture of SF_6_:O_2 _(20:4%) at a chamber pressure of 30 mTorr and RF power of 30 W. Followed by the dry-etching, samples were cleaned in a boiled acetone at 120°C for 5 min and in a methanol and finally rinsed in DI water (boiling temperature of acetone is 56°C). Based on the analyses, two test device structures were consequently fabricated using bare-Si and an ICP-etched Si using MWCNT dispersed in HMDS for a comparison of PS. An silicon on insulator (SOI) wafer was used for the device fabrication, which included 350 μm of *p*-type Si with a doping of 10^17 ^cm^-3^, 100 nm of SiO_2_, and another 100 nm thick *p*-type Si at the top with a doping of 10^17 ^cm^-3^. Both test structures were exactly the same except the active regions: bare-Si for one and ICP-etched Si for the other. For the contacts, Schottky-barrier of Cr/Ag (50/50 nm) was fabricated on the top layer using conventional photolithography and an e-beam evaporation at approximately 3 × 10^-5 ^Torr at approximately 150°C. For the measurements of surface morphology, an atomic force microscope (AFM, N8 ARGOS, Bruker AXS Inc.) was used in air with a non-contact mode. Reflectance was measured using Avaspec-3648 and halogen and deuterium lamps were used for light sources. For the *I*-*V *characterization, Keithley Semiconductor Characterization System (SCS-4200) was used under dark and at illumination (approximately 200 nm) with a power density of approximately 137 mW/cm^2^.

## Results and discussion

**Figure 1:**
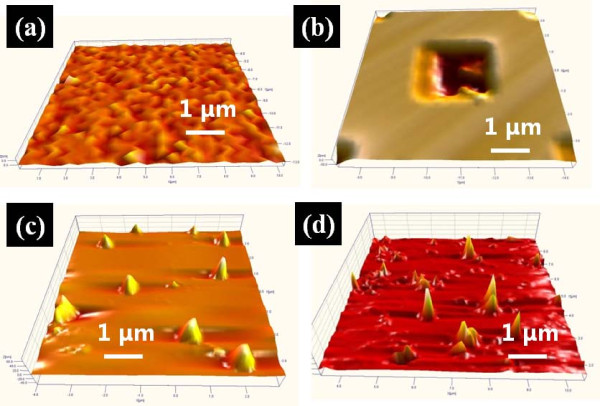
**(a) 3D AFM images of bare-Si**. **(b) **Conventional wet chemical etched Si using square masks. **(c) **ICP-etched Si surface using MWCNT-dispersion in isopropyl-alcohol. **(d) **ICP-etched Si using MWCNT-dispersion in HMDS. 3D AFM images in **(a-d) **are 7 (*x*) × 7 (*y*) μm^2^.

**Figure 2:**
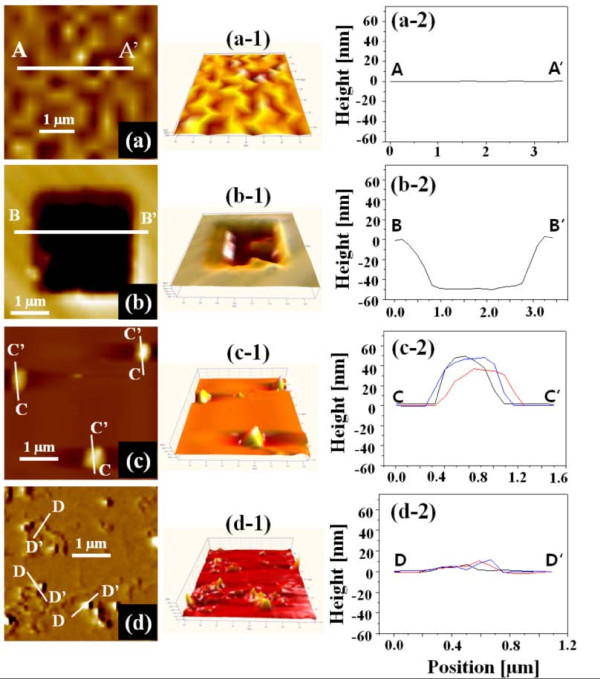
**2D and 3D views of AFM images and cross-sectional line-profiles of**: **(a)**. bare-Si **, (b) **conventional wet chemical etching of square-masked Si, **(c) **ICP-etched Si using MWCNT-dispersion in isopropyl-alcoholand **(d) **ICP-etched Si using MWCNT-dispersion in HMDS. Corresponding line-profilers are indicated as white lines in 2D AFM images with the corresponding alphabetical letters in **(a-d)**. 2D and 3D AFM images are 4 (*x*) × 4 (*y*) μm^2^.

Figure 1 shows three-dimensional (3D) AFM images of four Si samples with 7 (*x*) × 7 (*y*) μm^2 ^area. Figure 1a shows a bare-Si surface (sample A) and Figure 1b shows a square trench pattern on Si with an area of 3 × 3 μm^2 ^using conventional photolithography (sample B). MWCNT etch masked Si surface with the dispersion in isopropyl-alcohol (sample C) is presented in Figure 1c and 1 similarly MWCNT etch masked Si with a HMDS dispersion (sample D) is shown in Figure 1d. Figure 2 shows two-dimensional (2D) AFM top-views in the first column, 3D AFM side-views in the second column, and corresponding line-profiles in the third column of the representing structures of each sample. The *x*-axis in line-profiles represents the length and *y*-axis corresponds to the height along the line-profilers, which are indicated as white lines in the 2D AFM images. In all line-profiles, height (*y*-axis) is set to be equal in order for a clearer contrast. As seen in Figures 1a and 2a and a-1, sample A is fairly flat and there appears no distinctive structure with just micron-scale surface ripples. As clearly seen in the line-profile in Figure 2a-2, this surface is relatively very flat as compared to the other three. As seen in Figures 1b and 2b, b-1 and b-2, the sizes of trench squares are approximately 3 × 3 μm^2 ^at the top and approximately 2 × 2 μm^2 ^at the bottom. The square trench is approximately 50 nm deep as clearly seen in Figure 2b-2 and the bottom area of trench is roughened. As clearly seen in Figures 1c and 2c, c-1 and c-2, mound-like nano-structures appeared after an ICP etching, which can be likely due to an aggregation of MWCNTs [18-20] during the dry process. Average size of nano-mounds was approximately 45 nm in height and approximately 600 nm in length and average density of nano-mounds was 1.2 × 10^7 ^cm^-2 ^as seen in Figures 1c and 2c. With HMDS dispersion, the resulting surface after an ICP etching showed a much improved result as seen in Figures 1d and 2d and d-1. Line-like features can be clearly observed in Figures 1d and 2d, which was constructed by shadowing of MWCNTs during the ICP etching. Nevertheless, smaller mounds due to aggregation of MWCNTs were still observed and the size of nano-mounds was smaller ranging approximately 10-20 nm in height and approximately 200-400 nm in diameter.

**Figure 3:**
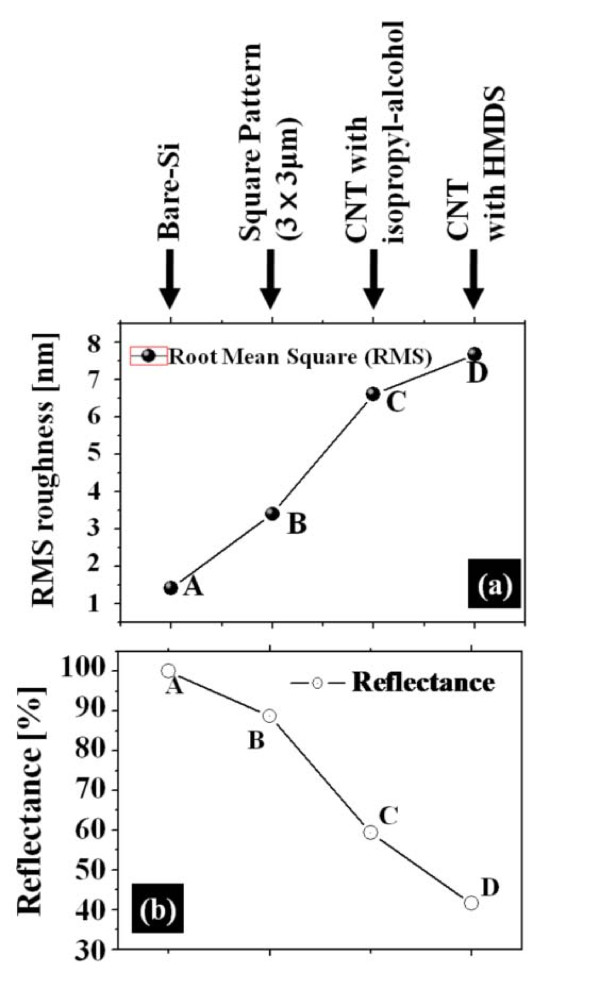
**(a) Plots of RMS roughness and (b) photo reflectanceof bare-Si, conventional wet chemical-etched Si using square masks, ICP-etched Si using MWCNT-dispersion in isopropyl-alcohol and ICP-etched Si using MWCNT-dispersion in HMDS**. The reflectance of bare-Si is set as a reference (100%). The spectral region is mid-UV in **(b)**.

**Figure 4:**
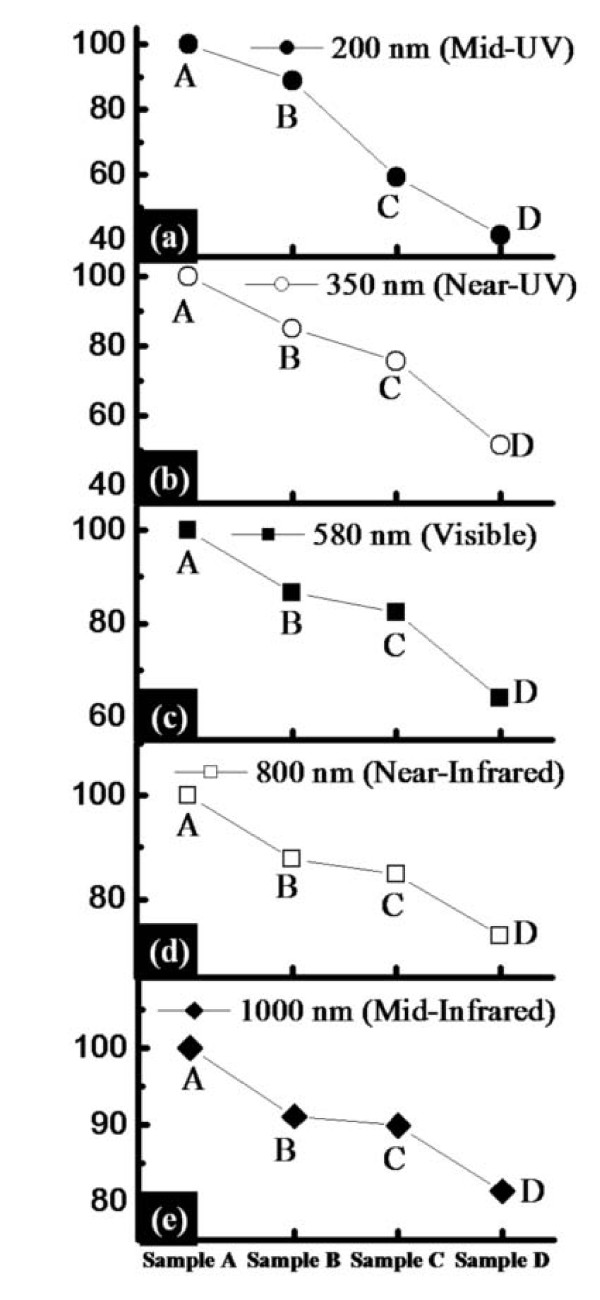
**Plots of photo reflectance versus samples over common spectral regions; mid-UV, near-UV, visible, near-infrared, and mid-infrared**. The decrease in photo reflectance is most significant at UV, less at visible, and finally somewhat insignificant at infrared.

Figure 3 presents root mean square (RMS) roughness (RMSR) analysis of sample A-D in Figure 3a and reflectance measurement in Figure 3b. *Y*-axis shows RMS roughness in nano-meter in Figure 3a and reflectance in % in Figure 3b. *X*-axis in Figure 3a, b indicates the four samples as labeled at the top of Figure 3a. The spectral region is mid-UV in Figure 3b. Overall, the RMSR analysis well matches with the AFM morphology analyses in Figures 1 and 2, i.e., the MWCNT etch-masked Si surface with HMDS dispersion (sample D) showed the highest RMRS and thus the roughest surface of the four. As can be expected from the AFM morphology analysis, sample D showed highest degree of roughness with an RMSR of 7.66 nm while sample A showed the lowest roughness with an RMSR of 1.41 nm. Sample B had an RMSR of 3.4 nm and sample C showed an RMSR of 6.56 nm, respectively. In comparison with the RMSR analysis, the reflectance acquired with an illumination at approximately 200 nm is plotted in Figure 3b and the reflectance of sample A (bare-Si) was set as 100% for a reference. Overall, the reflectance measurement also well matched with the AFM morphology and RMSR analyses. Namely, the sample D showed the lowest level of reflectance with a value of approximately 40%, indicating this surface possesses the highest light-trapping of the four samples. Meanwhile, the sample B showed a reflectance value of approximately 88% and sample C showed approximately 60%. Figure 4 shows the reflectance over common spectral regions versus samples, which includes UV, visible, and infrared. Over the 200-1000 nm spectral range, samples B-D showed a similar behavior: lowest reflectance at UV regions, somewhat higher at visible, and highest at infrared. This can indicate that the surface nano-structuring by ICP etching using MWCNT etch mask provides surface patterns that are sensitive to photons of shorter wavelengths. With a larger wavelength, the reflectance is expected to be less affected by the surface roughness as the wavelength becomes comparable with the surface features. Overall, the sample D showed the highest RMSR and lowest reflectance, especially significant at UV.

**Figure 5:**
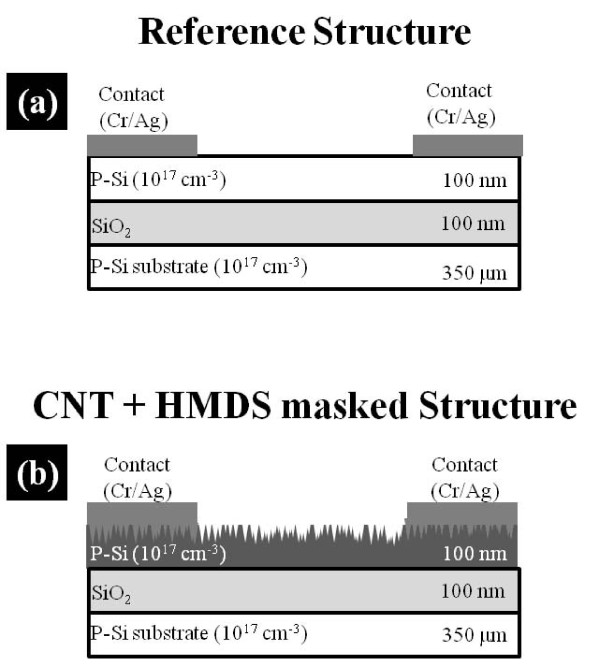
**Schematic descriptions of test device structures for *I*-*V *measurement**. **(a) **a reference device structure with an active region using bare-Siand **(b) **comparing device structure with an active region using ICP-etched Si with MWCNT dispersion in HMDS. Except the last P-Si layer, everything else is fabricated to be the same.

**Figure 6:**
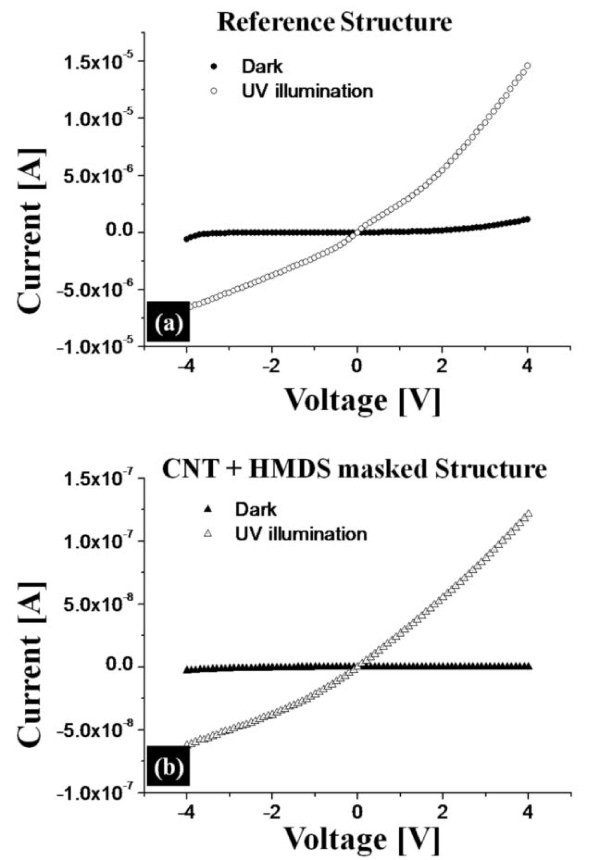
**Plots of *I*-*V *measurements under dark (black) and at UV illumination (white) of: (a)**. the reference device structure and **(b) **ICP-etched Si structure using MWCNT-dispersion in HMDS.

**Figure 7:**
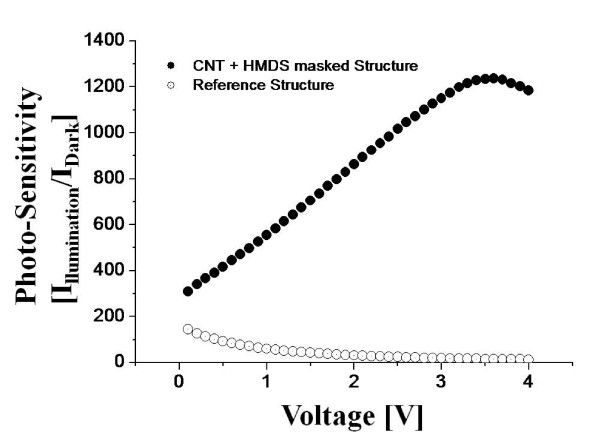
**Plot of PS indicating the ratio of the current at UV-illumination over the current under dark of the reference device and ICP-etched Si device**. ICP-etched Si device shows a much higher PS (black dots), indicating a much enhanced light-trapping of MWCNT masked Si under UV illumination.

In order to compare the efficiency of light-trapping, two device structures were fabricated. Figure 5 shows the two test device structures: reference structure using bare-Si in Figure 5a and MWCNT etch-masked Si with HMDS dispersion in Figure 5b. Both structures were fabricated for the same dimensions except the active regions (top layer). For the reference device, bare-Si with the highest reflection was used and for the other, MWCNT etch-masked Si with HMDS dispersion with the lowest reflection was used. For the contacts, Schottky-barriers of Cr/Ag (50/50 nm) were fabricated for both devices and the contacts were made after ICP etching for the MWCNT etch-masked device due to the CNT dispersion process. Figure 6 shows the *I*-*V *characteristic of both structures: reference device in Figure 6a and MWCNT etch-masked Si in Figure 6b. In Figure 6, black dots and triangles show dark current and white ones show current at UV illumination. At both cases, in general there appeared almost no (or very small) current under dark as can be expected. This shows that the current flow is regardless of the applied voltage under dark due to back-to-back Schottky-barriers, which is a common configuration of drain and source for SOI field-effect transistor. With an UV illumination, both devices showed sharp increases of current as voltage was increased. This can indicate that the optically generated carriers were swept down due to a tiled potential and the degree of the tilt is obviously as a function of an applied voltage. As clearly seen in Figure 6, the trends of current increase appear to be quire very similar. The current level with MWCNT etch-masked device is approximately two orders lower and this can be likely due to the fabrication of contacts after the ICP etching as mentioned above. In order to compare the effectiveness of light-trapping, PS of both devices is plotted in Figure 7. PS indicates a ratio of the current change at UV-illumination over the current under dark. Black dots indicate the PS of MWCNT etch-masked Si with HMDS dispersion while white ones are acquired from reference structure. In general, the CNT etch-masked device showed much enhanced PS, i.e., the ratio of UV-induced carriers was over 300 at 0.1 V and very sharply increased to over 1200 at 3.3 V. The increased PS can be due to the increased surface roughness and decreased reflectance and thus the increased light-trapping. Then the PS began to decrease at 3.3 V, indicating the maximum PS was approximately 1200. For the reference structure, the PS was initially somewhat higher ranging approximately 100 over 0.1-1 V and then decreased to several tens to approximately 10 over 1-3 V, indicating the PS was insignificant or the light was mostly reflected.

## Conclusions

In conclusion, to test light-trapping of Si, four samples were prepared: bare-Si, square masked-pattern, MWCNT etch-masked Si with isopropyl-alcohol dispersion, and MWCNT etch-masked Si with HMDS dispersion. The MWCNT etch-masked Si with HMDS dispersion (sample D) showed the height RMS roughness and lowest reflectance as compared to the other three tested Si samples. The reflectance was most significant at mid-UV region and less significant at infrared. Based on the RMS roughness and reflectance experiments, two device structures were fabricated with active regions of bare-Si and CNT etch-masked Si and tested under dark and at UV illumination. While both devices showed a similar behavior indicating increased current at UV illumination, the PS indicating the ratio of the current change at UV-illumination over the current under dark much sharply increased with the test device of CNT etch-masked Si. The increased photo response can be due to the increased surface roughness and decreased reflectance and thus the increased light-trapping. This result can find applications in such devices as photovoltaics, light-emitting diodes, photo-diodes, and photo-transistors, where the light-tramping is important.

## Abbreviations

AFM: atomic force microscope; CNT: carbon nanotube; HMDS: hexamethyl-disilazane; ICP: inductively coupled plasma; MWCNT: multi-walled carbon nanotube; PR: photo-resist.

## Competing interests

The authors declare that they have no competing interests.

## Authors' contributions

MYH & SMK participated in the experiment design, carried out the experiments. HSK, ESK, JHL, and SMK designed the experiments and testing methods. All authors helped to draft the manuscript and read and approved the final manuscript.
